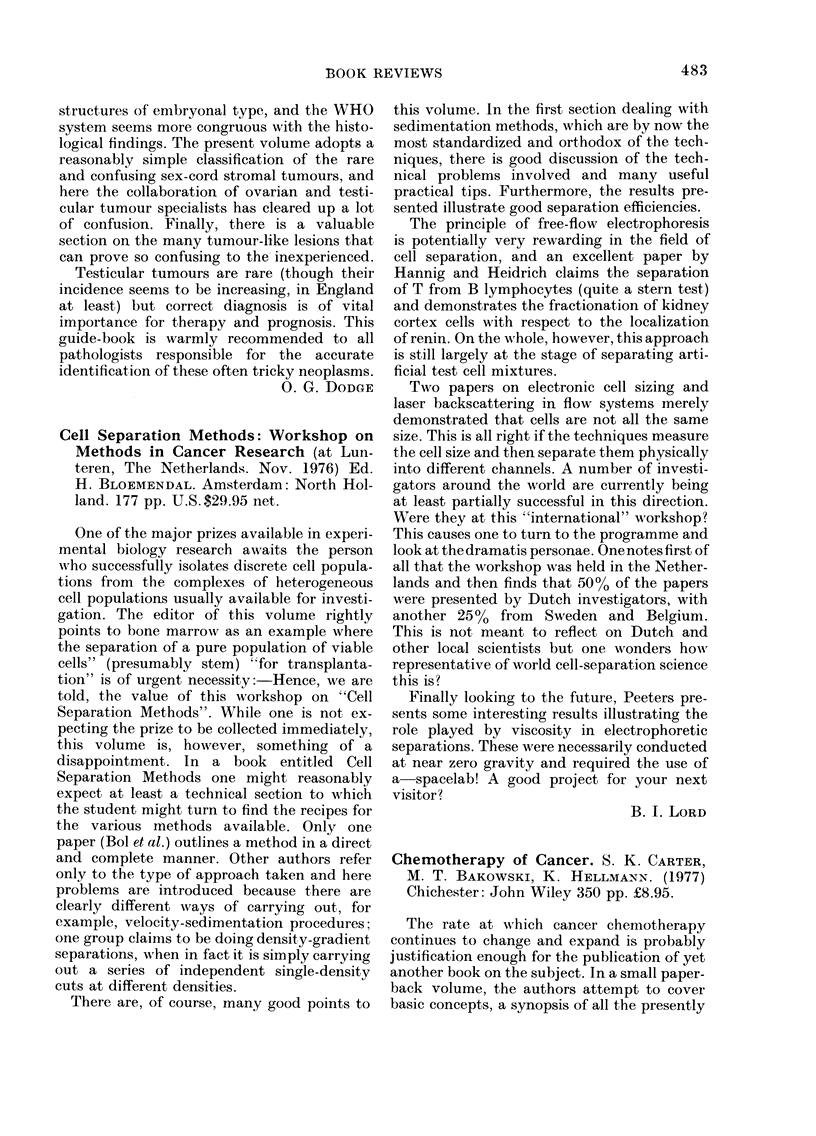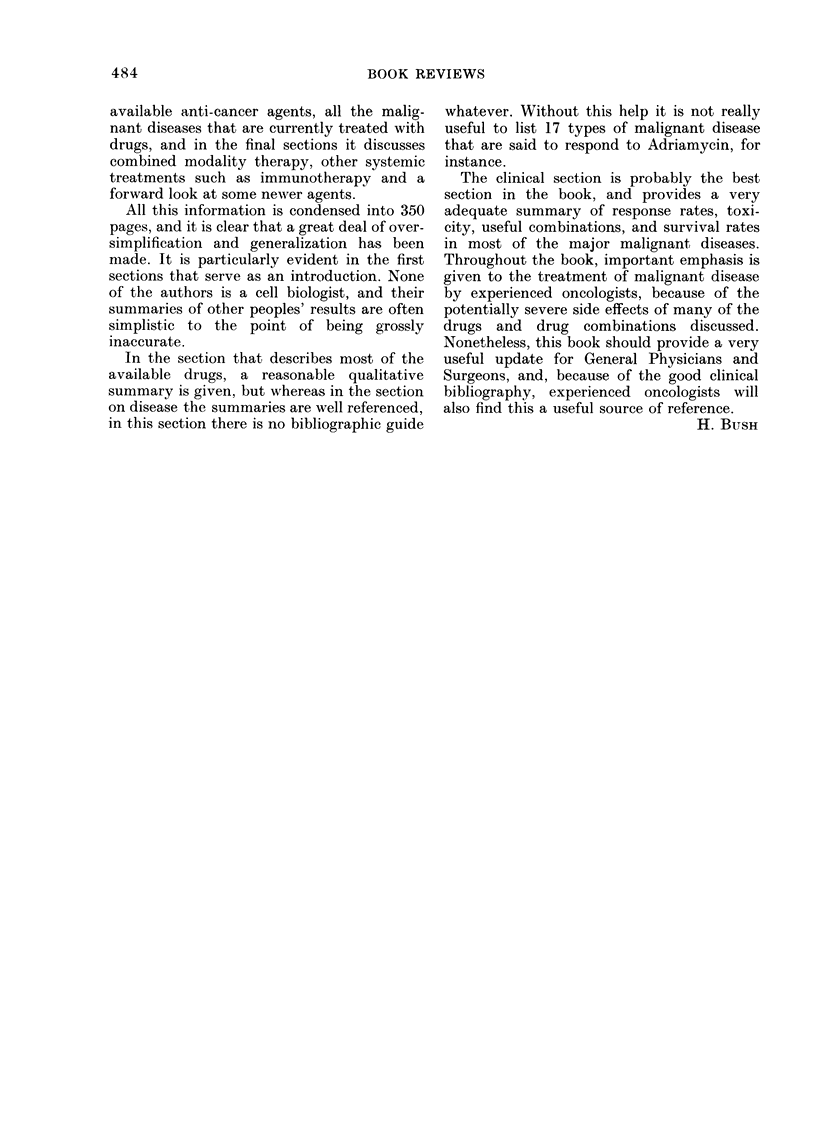# Chemotherapy of Cancer

**Published:** 1978-03

**Authors:** H. Bush


**Chemotherapy of Cancer.** S. K. CARTER, M. T. BAKOWSKI, K. HELLMANN. (1977) Chichester: John Wiley 350 pp. £8.95.

The rate at which cancer chemotherapy continues to change and expand is probably justification enough for the publication of yet another book on the subject. In a small paperback volume, the authors attempt to cover basic concepts, a synopsis of all the presently available anti-cancer agents, all the malignant diseases that are currently treated with drugs, and in the final sections it discusses combined modality therapy, other systemic treatments such as immunotherapy and a forward look at some newer agents.

All this information is condensed into 350 pages, and it is clear that a great deal of over-simplification and generalization has been made. It is particularly evident in the first sections that serve as an introduction. None of the authors is a cell biologist, and their summaries of other peoples' results are often simplistic to the point of being grossly inaccurate.

In the section that describes most of the available drugs, a reasonable qualitative summary is given, but whereas in the section on disease the summaries are well referenced, in this section there is no bibliographic guide whatever. Without this help it is not really useful to list 17 types of malignant disease that are said to respond to Adriamycin, for instance.

The clinical section is probably the best section in the book, and provides a very adequate summary of response rates, toxicity, useful combinations, and survival rates in most of the major malignant diseases. Throughout the book, important emphasis is given to the treatment of malignant disease by experienced oncologists, because of the potentially severe side effects of many of the drugs and drug combinations discussed. Nonetheless, this book should provide a very useful update for General Physicians and Surgeons, and, because of the good clinical bibliography, experienced oncologists will also find this a useful source of reference.

H. Bush